# Does Aerobic Exercise Facilitate Vaping and Smoking Cessation: A Systematic Review of Randomized Controlled Trials with Meta-Analysis

**DOI:** 10.3390/ijerph192114034

**Published:** 2022-10-28

**Authors:** Mohammad Z. Darabseh, James Selfe, Christopher I. Morse, Aseel Aburub, Hans Degens

**Affiliations:** 1 Al-Ahliyya Amman University, Department of Physiotherapy, Faculty of Allied Medical Sciences, Amman 19328, Jordan; 2 Manchester Metropolitan University, Department of Life Sciences, Research Centre of Musculoskeletal Sciences and Sport Medicine, Manchester M15 6BH, UK; 3 Manchester Metropolitan University, Department of Health Professions, Faculty of Health, Psychology and Social Care, Manchester M15 6BH, UK; 4 Manchester Metropolitan University, Department of Sport and Exercise Sciences, Research Centre of Musculoskeletal Sciences and Sport Medicine, Manchester M15 6BH, UK; 5 Keele University, School of Allied Health Professions, Keele ST5 5BG, UK; 6 Lithuanian Sports University, Institute of Sport Science and Innovations, 44221 Kaunas, Lithuania

**Keywords:** aerobic exercise, rehabilitation, smoking cessation, vaping cessation, exercise physiology, systematic review, meta-analysis

## Abstract

Cigarette smokers try to quit using several strategies including electronic cigarette use (vaping). An alternative, easy and cheap method is exercise. However, little is known about the efficacy of aerobic exercise (AE) to augment smoking and vaping cessation. This study aimed to systematically review and discuss the reported effects of AE on long-term vaping and smoking cessation in randomized control trials (RCTs). RCTs were searched on different databases. The outcome measures included long-term vaping or smoking cessation and maximal or peak oxygen uptake (VO_2max/peak_) after vaping- or smoking cessation. Meta-analysis was conducted to examine the effects of AE on long-term vaping and smoking cessation, and the effects of AE on VO_2max/peak_. Cochrane Risk of Bias tool 2 was used to assess trials quality. Thirteen trials were included (5 high, 2 moderate and 6 low quality). Although two high quality trials revealed that 3 vigorous supervised AE sessions a week for 12 to 15 weeks increased the number of long-term successful quitters, the meta-analysis including the other trials showed that AE did not significantly increase success rate of long-term quitters. However, VO_2max/peak_ was improved at the end of treatment. There were no trials on AE and vaping cessation. No evidence was found that AE promotes long-term smoking cessation. Nevertheless, AE improved VO_2max_ and/or VO_2peak_ in quitters.

## 1. Introduction

Smoking is considered the main risk factor for the development of preventable diseases such as cancers, cardiovascular diseases and respiratory disorders, including chronic obstructive pulmonary disease (COPD), and globally seven million deaths per year are attributable to smoking [1]. Smoking cessation (SC) reduces the risk of hospitalization due to chronic conditions, such as COPD, and is associated with significant life extensions [2,3]. As the annual death rate attributable to smoking is expected to increase within the next decades, the World Health Organization started calling upon governments and health institutes to develop anti-smoking regulations and interventions to further promote SC [1].

Although approximately 40% of smokers make at least one quit attempt annually [4], only fewer than 5% succeed [5]. Electronic-cigarette use (vaping) is promoted as a harmless and safe alternative to cigarette smoking [6] and uptake of vaping has been reported to be associated with higher rates of SC [7,8]. Vaping may, however, not be as harmless as originally thought and has been reported to cause similar detrimental effects on lung and cardiovascular function as smoking [9,10]. Such harmful effects may well contribute to the reportedly 33% of vapers that are willing to visit a vaping cessation service if available in their neighbourhood [11].

Beside vaping, SC interventions vary from pharmacotherapies including nicotine replacement therapy and SC counselling [12] to meditation and yoga programmes [13]. However, the success of these interventions is influenced by many factors such as the dose, type and duration of medication, intervention or counselling, motivational skills of SC advisors, follow-up periods, smokers’ adherence, duration of smoking and number of cigarettes one used to smoke per day. Indeed, the long-term effectiveness of these interventions remains ambiguous [14,15,16,17] and it is essential to keep looking for other interventions and assess their effectiveness.

One such potential alternative SC intervention is aerobic exercise. Exercise interventions are categorised as, e.g., aerobic, strengthening or relaxation exercises. As vaping and smoking particularly affect the cardiovascular and respiratory systems we consider here the impact of aerobic exercise training on the success of vaping and smoking cessation. In addition, it has been shown that aerobic exercise improves mood, well-being, and alleviates anxiety and depression, thereby contributing to the often-reported improvement in the quality of life [18,19,20,21,22]. Perhaps even more important is that exercise is easy to access and cheap and therefore one may consider exercise as a viable intervention to facilitate SC, particularly via the reduction in nicotine withdrawal symptoms and cigarette craving [23,24].

The mechanism by which aerobic exercise may enhance SC is not fully clear, but a number of mechanisms have been postulated, including raised endorphins, distraction and increased self-efficacy. It is known for example, that aerobic exercise induces an increase in plasma β-endorphins [25] that is dependent on the intensity and duration of the exercise performed [26]. The exercise-induced rise in β-endorphin levels may be significant as it has been found that higher levels were associated with fewer smoking relapses after cessation [27]. Additional mechanisms whereby aerobic exercise may facilitate SC are (1) increased proprioceptive input due to larger and more frequent movements that could distract smokers from cigarette craving [28] and (2) improved image self-efficacy [29]. Despite these potential mechanisms, the long-term benefits of exercise for smoking- and vaping- cessation are not clear.

When prescribing or describing exercise interventions, it is important to consider the frequency, intensity, time and type (FITT) of exercise [30,31,32]. The benefits of exercise training for vaping and smoking cessation may well depend on the duration, intensity and frequency of exercise training. As it is unclear whether aerobic exercise facilitates SC, a systematic review evaluating the effects of different exercise prescriptions (including the FITT principle) for vaping- and smoking cessation is warranted. Therefore, the aim of this review is to assess the effectiveness of aerobic exercise interventions on long-term vaping cessation and SC, and maximal and/or peak oxygen uptake. Where feasible this will be evaluated with meta-analyses.

## 2. Methodology

### 2.1. Purpose

The objectives were to review and discuss the reported effects of aerobic exercise on long-term vaping and smoking cessation, and to conduct a meta-analysis for the included trials.

### 2.2. Design

The study was designed to provide a systematic review with quality assessment, narrative synthesis, and meta-analysis of relevant published literature.

### 2.3. Study Protocol

The protocol of this systematic review is registered in the International prospective register of systematic reviews database (PROSPERO) (registration number: CRD42021232759; 2021).

### 2.4. Search Strategy

The following electronic databases were searched for trials published between 1 January 1970 to 19 January 2022: EBSCO host database including MEDLINE, AMED, SPORTDiscus and CINAHL; and PEDro. These databases were chosen because of the likely availability of exercise-related trials in these databases. Reference lists of included trials were hand searched to identify other potentially relevant trials. Trials included were limited to those written in English and published in peer-reviewed journals. Results of the searches were managed using Endnote Version X7 (Clarivate Analytics, Philadelphia, PA, USA).

### 2.5. Keywords

Search terms were adapted to meet the search requirements of each electronic database. The keywords used were structured according to the PICOS approach (population, intervention, comparison, outcome measures and study design) [33]. Table 1 summarizes the combinations of keywords included in the search strategies. PICOS search terms were combined using Boolean operators ‘AND’ and ‘OR’. The search was limited to randomised controlled trials (RCTs). To allow reproducibility of the search, the Medical Subject Headings (MeSH) were used.

### 2.6. Inclusion/Exclusion Criteria for the Trials

#### 2.6.1. Inclusion Criteria

Trials were included if:

- they included men & women > 18 years old
- they assessed continued/prolonged vaping cessation/smoking cessation by means of objective measures such as carbon monoxide (CO), cotinine and/or thiocyanate level
- participants had been smoking for ≥6 months and smoked/smoke ≥5 cigarettes per day or vaped for ≥6 months

#### 2.6.2. Exclusion Criteria

Trials were excluded if:

- the intervention was other than cardiovascular/aerobic exercise, or if the aerobic exercise was combined with another type of exercise
- the exercise type used was not identified
- the outcome measures did not include CO, cotinine and/or thiocyanate
- the period of vaping/smoking cessation was less than six months
- not written in English language
- participants were diagnosed with psychiatric illness that could affect their exercise adherence (for example: depression or anxiety)
- there were substance misuse problems (such as drugs and alcohol abuse)
- participants were pregnant
- participants suffered from any medical condition that might affect their exercise performance such as musculoskeletal or neurological conditions
- published protocols were presented but without published data/results, or if they were conference abstracts

### 2.7. Study Selection

The first reviewer (MD) retrieved all trials from initial database searches and imported these into Endnote software. Trials were screened for suitability by the first reviewer (MD) by consulting the title and abstract against the pre-defined eligibility criteria for potential full-text review. The second reviewer (AA) independently screened the trials by consulting titles and abstracts against the pre-defined eligibility criteria for potential full text review.

### 2.8. Risk of Bias and Quality Assessment of the Included Trials

Risk of bias of included trials was assessed using the Cochrane Risk of Bias tool 2 (CROB 2). Two review authors independently assessed the risk of bias. The following were assessed using the CROB 2: (1) bias arising from the randomization process; (2) bias due to deviations from intended interventions; (3) bias due to missing outcome data; (4) bias in measurement of the outcome; (5) bias in selection of the reported result.

Two review authors independently assessed the quality of the included trials using the PEDro Scale, a validated tool for assessment of quality of interventional trials specifically related to physiotherapy interventions [34,35]. The PEDro scale contains 11 items, and trials are awarded between 0 and 10 points, depending on the number of criteria they meet (the first item is not used to calculate the summary score). Trials with scores of four points or more are classified as “high-quality”, whereas trials with three points or fewer are classified as “low-quality” [34,35]. PEDro and CROB 2 scores for the trials were not used as inclusion or exclusion criteria, but as a basis for best-evidence synthesis and to determine the strengths and weaknesses of each trial.

### 2.9. Data Extraction

The following data were extracted from the included trials: author name(s); year of publication; sample size; age; intervention for each group; outcome measures; comparator group; duration of the follow-up period; number of participants at baseline; number of participants who remained abstained at the final follow-up period; intervention for each group, including exercise prescription component (frequency, intensity, time and type of exercise); the physiological effect of aerobic exercise on cessation (e.g., increases in maximal and/or peak oxygen uptake) after vaping/smoking cessation.

Extracted data were consulted and checked with the second reviewer (AA).

### 2.10. Outcome Measures

The main outcome measure was the proportion of participants who successfully quit vaping or smoking for at least six months, verified by objective measures such as CO, cotinine and/or thiocyanate concertation at the last/longest period of assessment (follow up).

Where reported, the physiological effect of aerobic exercise was included in the review, e.g., increases in maximal and/or peak oxygen uptake (VO_2max/peak_) after vaping/smoking cessation.

### 2.11. Measurement of Treatment Effect

The risk ratio (RR) was calculated as = (quitters in exercise group/total randomised to exercise group)/(quitters in control group/total randomised to control group), with a 95% confidence interval (CI). Where more than one exercise group was included, the sum of the participants in all exercise groups was compared with the sum of all participants in all control (non-exercise) groups.

Standardised mean differences and their 95% CI were calculated from the data generated by each included randomised controlled trial for VO_2max_ or VO_2peak_ results. Forest plots were used to present the effectiveness of exercise on vaping- and smoking cessation, and the effects of aerobic exercise on VO_2max_ or VO_2peak_, using the OpenMetaAnalyst software.

Where statistical pooling was not possible, the findings were presented in narrative form.

### 2.12. Dealing with Missing Data

All data that were available in the included trials were included in the Meta-analysis with intention-to-treat.

### 2.13. Heterogeneity Assessment

After pooling data from the trials, statistical heterogeneity was determined using the I^2^ statistic [36]. I^2^ < 50% indicates low heterogeneity.

## 3. Results

### 3.1. Results of the Search

**Figure 1:**
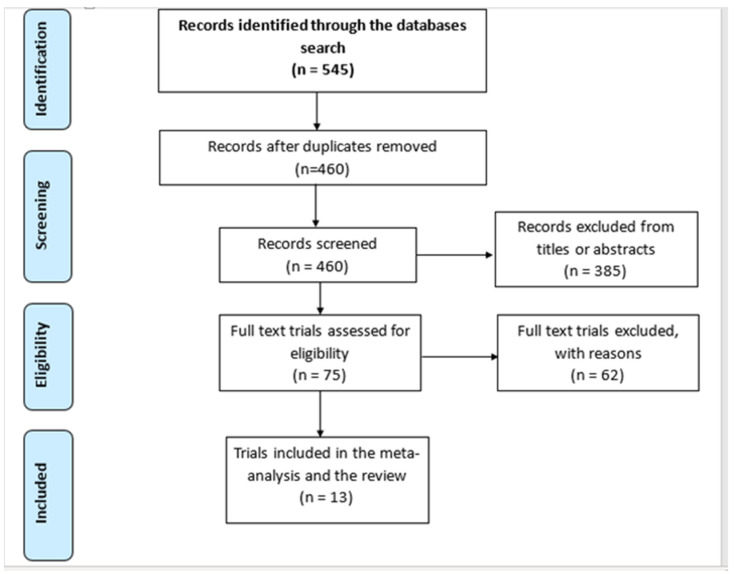
The PRISMA flow-chart for the search records and the included trials.

The systematic search identified 545 articles, 85 of which were duplicates. After screening the titles and abstracts, 385 publications were considered not relevant. Of the 75 remaining trials, 62 were excluded: 10 were using combined exercises, or combined exercise and diet management; 3 included participants with diagnosed depression; 11 did not include objective smoking cessation measures (such as CO, cotinine, or thiocyanate); 28 did not include aerobic exercise or did not specify the type of exercise used; in 5 the follow up on the effects of aerobic exercise was <6 months; 3 aimed for smoking reduction not cessation; 1 only conducted exercise counselling but not exercise and 1 trial presented preliminary results for an already included full trial. Consequently, 13 trials were included. Figure 1 shows the Preferred Reporting Items for Systematic Reviews and Meta-Analyses (PRISMA) flow-chart for the included/excluded search records. Table 2 is the data extraction table for the included 13 trials. No disagreement was encountered between the first and second reviewer in study selection.

### 3.2. Risk of Bias and Quality Assessment

**Figure 2:**
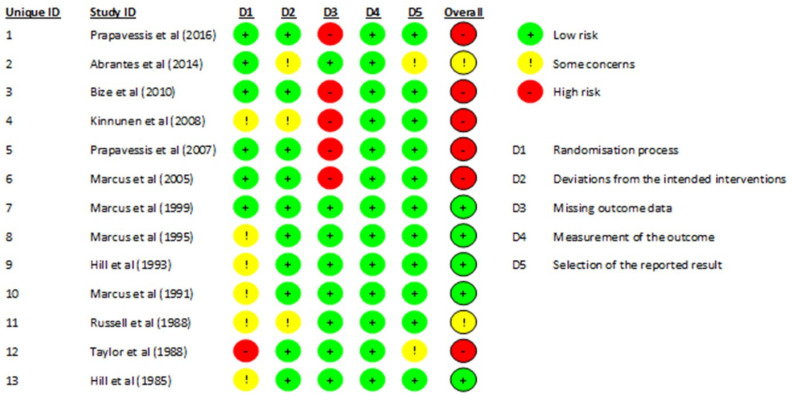
Results of the Cochrane Risk of Bias (CROB2) for the included trials. References cited in the figure are: [37,38,39,40,41,42,43,44,45,46,47,48,49].

Five trials were at low risk of bias (low risk of bias across all domains, or low risk of bias in four domains and one domain with “some concern”), six trials were at high risk of bias (high risk of bias in at least one domain), and the remaining two at unclear/some concern of risk of bias. A summary of the CROB2 results is shown in Figure 2.

The PEDro scale results revealed that the included trials were of high quality (total PEDro score > 4 points), with most of the trials scoring 6 points. Only one trial scored 5 [37], as groups were not similar at baseline (Table 3). Three trials scored 7 [38,39,40], because allocation of participants was concealed. No disagreement was encountered between the first and second reviewer in terms of risk of bias assessment.

**Table 2:** Data extraction table for the included trials.

Author (Year)	Sample Size (n)	Age Mean (SD) in Years	M:W (n)	Intervention/s (for Each Group) FITT (Where Possible)	Outcome Measures	Key Findings
[41]	61 (total) G1 = 30 G2 = 31	Total = 47.3 G1 = 47.1 (8.5) G2 = 47.5 (10.7)	21:40	**G1:** 12-week group supervised exercise intervention (12 one session a week) + Telephone counselling SC intervention that included TNP (8 sessions, weekly, 20 min each) + exercise group counselling/discussion weekly (for 12 weeks, for 20 min) + unsupervised aerobic exercise sessions. Exercise began before quitting date **G2:** 12 weeks SC counselling sessions: (12 sessions, 1 h each) + Telephone counselling SC intervention that included TNP (8 sessions, weekly, 20 min each). **For exercise prescription:** **F:** Once a week exercise supervised session + two to four unsupervised exercise sessions a week. **I:** Moderate exercise (range of 55–69% of age-predicted HRmax). **T:** Began at 20 min per session with weekly gradual increases, to 100 min midway through the intervention up to 150 min towards the ends of the intervention. **T:** Treadmill, stationary bicycles, walking, running, sports, cycling and housework	Assessments occurred at baseline, 3(EOT), 6, and 12-month follow-ups. SC Self-reports verified by expired CO; utilizing 10 ppm cut-off at each assessment timepoint. VO_2peak_ treadmill test.	No significant difference in abstinence between groups (*p* = 0.18). Participants in G1 had higher verified cessation rates (EOT: 30.0% in G1 vs. 25.8% in G2), and 12-month follow-up (13.3% in G1 vs. 3.2% in G2). VO_2peak_ was increased similarly in both groups: G1: baseline = 27.8 (5.8) mL/kg/min, EOT = 30.0 (5) mL/kg/min G2: Baseline = 26.2 (9.6) mL/kg/min, EOT = 27.3 (6) mL/kg/min. At EOT, adherence in both G1 and G2 was 9.3 ± 2.8 vs. 9.3 ± 3.0 out of the 12 sessions, respectively.
[38]	481 (total) G1 = 229 G2 = 252	Total = 42.2 (10.1) G1 = 42.2 (10.0) G2 = 42.5 (9.5)	272:209	**G1:** 9-week exercise group supervised intervention (9 sessions-once a week) + 15 min individual based SC intervention and counselling sessions weekly (for 9 weeks) including NRT products prescription such as TNP, gum, inhaler and lozenge + unsupervised exercise sessions. Exercise started 1 week before quitting date **G2:** 9-weeks SC individual based SC intervention weekly (9 sessions) for 15 min session including NRT products prescription such as TNP, gum, inhaler and lozenge + 9-weeks 60 min supervised group sessions health education (discussions, lectures etc). **For exercise prescription:** **F:** One supervised exercise session a week + four unsupervised (home based) sessions a week **I:** Moderate exercise (intended to target 40–60% of maximal aerobic power) **T:** 45 min per session supervised and 30 min unsupervised exercise sessions. **T:** Brisk walking and slow jogging, commuting on foot or by bicycle, leisure/recreational and aerobic housework activities.	Follow-up at 10, 26 and 52 weeks after the beginning of the SC programme SC Self-reports verified by expired CO; utilizing 10 ppm cut-off at each assessment time point. The intensity of physical activity was monitored with the Borg Rating of Perceived Exertion Scale	Participation in a weekly population-based programme of moderate-intensity physical activity for 9 weeks was not sufficient to increase SC rate when added to a comprehensive SC programme offering individual counselling and NRT. Continuous cessation rates were high and similar in G1 and the G2 at the EOT (47% vs. 46%, *p* = 0.81), and similarly decreased at 26 weeks (34% vs. 35%, *p* = 0.77) and at 1-year follow-up (27% vs. 29%, *p* = 0.71), respectively. At 52-weeks follow-up, the adherence in G1 was 55% and in G2 62%.
[42]	36 (total) G1 = 18 G2 = 18	Total = 40 G1 = 37.67 (8.77) G2 = 41.61 (7.59)	10:26	**G1:** 5-week supervised (if participants’ circumstances allowed, if not, they were asked to do unsupervised sessions) exercise intervention (10 sessions-twice a week) + group SC counselling sessions twice a week (for 5 weeks) for 60–90 min per session + unsupervised exercise sessions Exercise began on the quitting date. **G2:** 5-weeks group SC counselling intervention twice a week (total of 10 sessions) for 60–90 min per session **For exercise prescription:** **F:** Twice a week group session + as often as possible times unsupervised sessions a week **I:** Not specified **T:** 30 min per session supervised and as long as possible unsupervised **T:** Bicycle ergometer, walk or jog, bicycle ride, running, walk up and down of stairs	Assessment occurred at baseline, 5 weeks (EOT), follow-up: 1, 3, and 6 months SC Self-reports verified by expired CO; utilizing 10 ppm cut-off at each assessment time point. VO_2max_ cycle ergometer	No significant difference in quit rate between G1 and G2 (*p* = NS) G1 VO_2max_ significantly increased from 30.28 mL/kg/min at baseline to 32.11 mL/kg/min at EOT (*p* < 0.05), compared to G2 who have slight increase from 30.52 mL/kg/min to 30.9 mL/kg/min (*p* = NS).
[43]	82 (total) G1 = 22 G2 = 22 G3 = 18 G4 = 20	Total = 59	39:43	**G1:** Behavioural treatment only **G2:** Behavioural treatment combined with nicotine gum **G3:** Behavioural treatment combined with supervised or unsupervised physical exercise **G4:** Supervised or unsupervised physical exercise **For G3 and G4 exercises prescription:** **F:** 3 Supervised or unsupervised sessions a week for 12 weeks **I:** 60–70% of HR reserve **T:** 45 min **T:** graduated walking (indoor and outdoor)	Quit rates were assessed at EOT and at 4, 7, and 12 months as follow up sessions SC Self-reports verified by expired CO; utilizing 10 ppm cut-off at each assessment time point.	At 12 months the proportion of quitting across groups were (G1 = 31.8%, G2 = 36.4%, G3 = 27.8%, and G4 = 10.0%) indicating that behavioural training facilitated cessation (G1, G2 and G3) better than the physical exercise only (G4) (*p* < 0.01). The adherence rates were: G1 65%; G2 66%; G3 57% and G4 53%.
[44]	182 (total) G1 = 92 G2 = 56 G3 = 34	Total = 39 G1 = 38.3 (9.9) G2 = 37.9 (9.1) G3 = 39.9 (9.9)	0:263	**G1:** Aerobic exercise supervised sessions + SC counselling sessions (once a week for 19 weeks) + nicotine gum + home based exercise sessions (e.g., walking, exercise tapes) to bring their total number of weekly exercise sessions to at least three. **G2:** SC counselling sessions (once a week for 19 weeks) + nicotine gum + health education sessions and discussions **G3:** SC counselling sessions (eight session over the 19 weeks) + nicotine gum Participants were followed from 3 weeks before cessation to 1year post cessation. **For G1 exercise prescription:** **F:** Twice a week for 5 weeks, then once a week for 14 weeks + home based exercise sessions (30 min) to bring their total number of weekly exercise sessions to at least three **I:** 60–80% HRmax **T:** 40 min **T:** Walking or running on a treadmill	Quit rates were assessed at EOT and at follow-up: 1 week, 1, 4 and 12 months SC Self-reports verified by expired CO; utilizing 10 ppm cut-off at each assessment time point and salivary cotinine levels. VO_2max_ treadmill test	G1 and G2 at EOT and 12 months follow-up had a similar rate of cessation as G3 (*p* = NS) The increase in VO_2max_ from baseline to EOT was significantly higher in G1 than G2 and G3 (*p* < 0.05): G1: baseline = 28.8 (8.5) mL/kg/min, EOT = 32.9 (7.7) mL/kg/min G2: baseline = 28.0 (4.2) mL/kg/min, EOT = 30.1 (2.9) mL/kg/min G3: baseline= 34.2 (5.8) mL/kg/min, EOT = 35.3 (6.9) mL/kg/min. The combined pre and post cessation adherence rates were higher in G2 (85%) than in G1 (74%) (*p* < 0.001).
[45]	20 (total) G1 = 10 G2 = 10	Total = 39 (8) G1 = 40 (9) G2 = 38 (8)	0:20	**G1:** Aerobic exercise group supervised sessions + SC counselling sessions (twice a week for 4 weeks) **G2:** SC counselling only (twice a week for 4 weeks) Exercise began before quitting date **For G1 exercise prescription:** **F:** 3 sessions supervised exercise sessions a week for 15 weeks **I:** 70–85% HRmax **T:** 30–45 min **T:** cycle ergometry and treadmill walking	Quit rates were assessed at EOT and at follow-up: 1, 3, 12 months. SC Self-reports verified by saliva cotinine < 10 ng/mL VO_2max_ cycle test	Four participants in G1 remained abstinent at 1 month, 3 participants at 3 months and 2 participants at 12 months after SC treatment, compared with zero in G2 (*p* < 0.05). Only in G1 VO_2max_ was increased (*p* < 0.01) G1: baseline = 26 (6) mL/kg/min, EOT = 31 (3) mL/kg/min G2: baseline = 26 (5) mL/kg/min, EOT = 26 (2) mL/kg/min (No increase nor decrease). Adherence rate was only mentioned for G1 and was 88% of the sessions.
[46]	20 (total) G1 = 10 G2 = 10 Contact control	38 (total) G1 = 36 (10) G2 = 39 (8)	0:20	**G1:** Aerobic exercise group supervised + SC counselling sessions (once a week for 12 weeks) **G2:** SC counselling sessions (once a week for 12 weeks) + health education 3 times a week (for 45 min each) for 12 weeks Exercise began before quitting date **For G1 exercise prescription:** **F:** 3 sessions a week supervised exercise sessions for 12 weeks **I:** 70–85% HRmax **T:** 30–40 min **T:** cycle ergometry and treadmill walking	Quit rates were assessed at EOT and at follow-up: 1, 3, 12 months. SC Self-reports verified by expired CO (utilizing 8 ppm cut-off at each assessment time point) and saliva cotinine < 10 ng/mL VO_2max_ cycle test	There were no significant differences at EOT in favour of the G1 over G2 (4 vs. 2 participants). At 1 and 3 months follow-up, the same four G1 participants remained abstinent. At the 12-month follow-up, three of G1 participants remained abstinent. One participant only in G2 remained abstinent All three participants of G1 who were abstinent at 12 months had continued exercising. The increase in VO_2max_ was higher in G1 than G2 (*p* < 0.05): G1: baseline = 24 (4) mL/kg/min, EOT = 30 (4) mL/kg/min. G2: baseline = 28 (6) mL/kg/min, EOT = 27 (1) mL/kg/min. At EOT, adherence rate: G1: 85% of the smoking cessation sessions; 88% of the exercise sessions G2: 85% of the smoking cessation sessions; 92% of the contact sessions.
[47]	281 (total) G1 = 134 G2:147	G1= 40.7 (9.1) G2= 29.7 (8.8)	0: 281	**G1:** Aerobic exercise groups supervised sessions + SC counselling sessions (once a week for 12 weeks). **G2:** SC counselling sessions (once a week for 12 weeks) + health education (45–60 min each) 3 times a week for 12 weeks. Exercise began before quitting date **For G1 exercise prescription:** **F:** 3 sessions a week supervised exercise sessions for 12 weeks **I:** Vigorous 60–85% HR reserve **T:** 40–50 min **T:** cycle ergometry and treadmill walking	Quit rates were assessed at EOT and at follow-up: 3, 12 months. SC Self-reports verified by expired CO (utilizing 8 ppm cut-off at each assessment time point) and saliva cotinine < 10 ng/mL VO_2peak_ cycle test	G1 participants were more likely than G2 participants to be continuously abstinent during the 8 weeks of treatment following quit day (19.4% vs. 10.2%, *p* = 0.03). G1 participants were more likely than G2 participants to achieve 3 and 12 months of continuous abstinence following quit day (3 months: 16.4% vs. 8.2%, *p* = 0.03; 12 months: 11.9% vs. 5.4%, *p* = 0.05). The increase in VO_2peak_ was higher in G1 than G2 (*p* < 0.01): G1: baseline = 25 (6) mL/kg/min, EOT = 28 (6) mL/kg/min. G2: baseline = 25 (5) mL/kg/min, EOT = 25 (5) mL/kg/min (No increase nor decrease). At EOT, adherence rate for G1 was 68.7%, and for G2 64.6%. At 12 months follow-up, adherence rate for G1 was 56% and for G2 50.3%.
[40]	217 (total) G1 = 109 G2 = 108	G1 = 42.52 (10.4) G2 = 43.02 (10.3)	0:217	**G1:** Aerobic exercise groups supervised sessions + home based exercise 4 times a week for 30 min each + SC counselling sessions (1 h, once a week for 8 weeks). Offered nicotine patch. **G2:** SC counselling sessions (1 h, once a week for 8 weeks) + health education (1 h, once a week for 8 weeks). Offered nicotine patch. Exercise began before quitting date **For G1 exercise prescription:** **F:** One session a week for 8 weeks **I:** Moderate, 45–59% HR reserve or 50%–69% of HRmax **T:** 55 min **T:** cycle ergometry and treadmill walking	Quit rates were assessed at EOT and at follow-up: 3, 12 months. SC Self-reports verified by expired CO (utilizing 8 ppm cut-off at each assessment time point) and saliva cotinine < 10 ng/mL Functional capacity expressed as VO_2peak_ treadmill test	No significant differences between G1 and G2 at EOT and 3 months follow up (14.7% and 7.3% for G1 vs. 11.1% and 3.7% for G2, *p* = NS, respectively). No group differences were found at 12 months follow up of continues cessation (0.09% for G1 vs. 0.09% for G2, *p* = 0.75), where both groups were equally likely to report SC at EOT The increase in VO_2max_ was significantly higher in G1 than G2 (*p* < 0.05): G1: baseline = 30.71 (6.12) mL/kg/min, EOT = 31.88 (6.35) mL/kg/min G2: baseline = 30.68 (5.67) mL/kg/min, EOT = 30.4 (5.62) mL/kg/min. At EOT, adherence for G1 was 54.1% and for G2 58.9%. At 12 months follow up, adherence for G1 was 24.8% and for G2 31.8%.
[48]	142 (total) Phase 1: G1 = 76 G2 = 66 Phase 2: G1 = 35 G2 = 33 G3 = 27 G4 = 26	Total = 38 G1 = 37.9 (12.4) G1 = 38.2 (10.9)	0: 142	**Phase 1:** 6 weeks **G1:** Supervised exercise programme **G2:** Supervised cognitive behavioural SC programme (3 times a week for 5 weeks) **Phase 2:** 7–12 weeks, 121 participants who made a quit in phase 1, were randomised to 1 of 4 groups in phase 2: **G1:** Aerobic group exercise + SC counselling (3 times a week for 6 weeks) **G2:** Aerobic group exercise + nicotine patches **G3:** Cognitive behavioural cessation programme (3 times a week for 6 weeks) **G4:** Cognitive behavioural cessation programme (3 times a week for 6 weeks) + nicotine patches. Exercise began before quitting date **For exercise prescription:** **F**: Three times a week for 12 weeks **I**: 60–75% HR reserve **T**: 45 min **T**: Cycle ergometry, treadmill and rower	Quit rates were assessed at EOT and at follow-up: 3, 12 months SC Self-reports verified by expired CO (utilizing 10 ppm cut-off at each assessment time point) and saliva cotinine < 10 ng/mL Physical work capacity (PWC 75%) cycle ergometer test	For continuous abstinence, no significant differences between groups were noted at the three post-quit time periods. At 3-month follow-up and 12-month follow-up, 33.9% and 22.0% of those who received patches compared to 25.8% and 11.3% of those who did not receive patches remained continuously abstinent, respectively (*p* = 0.33; *p* = 0.11). At EOT, participants who received the nicotine patches (irrespective of group) were more likely to remain abstinent (72.9% vs. 53.2%) (*p* = 0.03). At EOT, G1 had significantly increased their PWC compared to G2 (*p* < 0.01) At EOT, adherence for G1 + G2 was 62.4% of the exercise sessions and for G3 + G4 62.8% of their smoking cessation sessions.
[39]	413 (total) G1 = 108 G2 = 106 G3 = 100 G4 = 95	G1 = 41.96 (12.7) G2 = 43.47 (14.0) G3 = 43.45 (12.2) G4 = 40.36 (11.9)	0:413	Participants completed a 14-week exercise programme with NRT (TNP). NRT started after 4 weeks of exercising. Then, randomised to 1 of 4 groups **G1:** Exercise maintenance (group supervised) + SC maintenance **G2:** Exercise maintenance (group supervised) + contact control **G3:** SC maintenance+ contact control **G4:** Contact control G1 + G2 during weeks 8–14 received cognitive behavioural therapy sessions in groups, five sessions a week for 25 min with the goal of teaching self-regulatory skills and for exercise adherence. Additionally, during weeks 26 and 52 they received telephone counselling seven sessions for 15 min biweekly (for the first month), then monthly (for the next 2 months) and then bimonthly (for last 8 months). G3 + G4 contacted by messages reinforcing women’s health issues. Additionally, during weeks 26 and 52 they were contacted by messages reinforcing the Forever Free booklets and/or women’s health issues. **For exercise prescription:** **F:** First 8 weeks three sessions a week, weeks 9–11 two sessions a week and weeks 12–14 only one session + unsupervised at weeks 8–14 three sessions a week similar to the supervised duration and intensity. **I:** 70–75% HRmax **T:** 45 min supervised. 15 min unsupervised **T:** Treadmills, rowing machines, stair climbers and stationary bicycles	Quit rates were assessed at EOT and at follow-up: EOT (week 14), 26, 56 weeks SC Self-reports verified by expired CO (utilizing 6 ppm cut-off at each assessment time point)	At week 26, there was no significant difference in the proportion of abstainers (*p* = 0.77) At week 56, there were no significant differences in the cessation rates between G1 (32.8%), G2 (19%), G3 (27.6%) and G4 (20.7%) (*p* = 0.43) At EOT, adherence G1 was 50.93%; G2 53.15%; G3 49.33% and G4 45.26%.
[49]	42	Total = 28 (7)	0:42	One week (4 1 h sessions) behavioural smoking cessation program, then randomly assigned into: **G1:** Group aerobic exercise class sessions + home based (2 sessions) **G2:** Group SC counselling including health education (1 h each, 9 sessions) **G3:** Control group (reports weight, CO and withdrawal symptoms) Exercise began after quitting date **For exercise prescription:** **F:** 3 sessions a week (one supervised and two unsupervised) for 9 weeks **I:** 70–80% HRmax **T:** 20–30 min **T:** Cycling, walking, jogging and home-based aerobic exercises.	Quit rates were assessed at EOT and at follow-up: 3, 6 months SC Self-reports verified by expired CO PWC 150 cycle ergometer test	EOT cessation rates were high (83% irrespective of group) for all groups at the end of the program There were no significant differences in cessation across groups; the cessation rates were decreased from 83% at the EOT to 73% at 3 months, 49% at six months and 34% at 18 months for all groups.
[37]	203 initially and ended up with 68 G1 = 42 G2 = 26	Total = 52 (9)	68:0	Started as **G1:** Supervised aerobic exercise followed by home based exercise training (54) + one SC counselling session (at week 3 post AMI) **G2:** Supervised aerobic exercise followed by medically supervised group exercise training (53) + one SC counselling session (at week 3 post AMI) **G3:** Supervised aerobic exercise only (26) + one SC counselling session (at week 3 post AMI) **G4:** Control (Participants were seen for the first time at 26 weeks for aerobic exercise testing) (27) **Ended up as** G1 + G2 pooled to be exercise group G3 + G4 pooled to be non-exercise group **For exercise prescription:** Not available in the text.	Quit rates were assessed at EOT and at follow-up: 26 weeks SC Self-reports verified by plasma thiocyanate, utilizing 100 mmol/L as cut-off Functional capacity treadmill peak test	12% (5/42 participants) in the G1 and 1% (5/26 participants) in G2 were still smoking at 3 weeks. None of the 10 participants who were smoking at 3 weeks stopped by 26 weeks (*p* = NS) By 23 weeks, cessation rates were 69% (29/42) in G1 and 61% (16) in G2, respectively. Between week 3 and 26 significant improvement in VO_2peak_ level in exercise groups compared to non-exercise group (average increase of 6.65 mL/kg/min vs. 4.2 mL/kg/min, respectively (*p* < 0.05)).

G1: Group 1; G2: Group 2; G3: Group 3; G4: Group 4; M: Man; W: Women; SC: Smoking cessation; NS: Not significant; FITT: Fitness, Intensity, Time, Type; VO_2max_: maximum oxygen uptake; VO_2peak_: peak oxygen uptake; TNP: Transdermal nicotine patch; EOT: End of treatment; CO: Carbon monoxide; NRT: Nicotine replacement therapy; HR: Heart rate; PPM; parts per million; PWC: Physical work capacity; AMI: Acute myocardial infarction.

## 4. Meta-Analysis Results

### 4.1. Effectiveness of Aerobic Exercise to Facilitate Smoking Cessation

**Figure 3:**
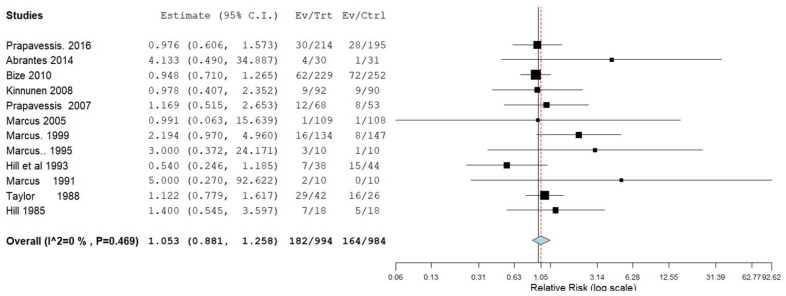
Forest plot for the success of Smoking cessation (SC). CI: confidence interval; Ev: number of quitters in the exercise group/s at the last follow-up session; Trt: number of participants in the exercise group at baseline; Ctrl: number of participants in the non-exercise group/s at baseline session. References cited in the figure are: [37,38,39,40,41,42,43,44,45,46,47,48].

To assess the effectiveness of aerobic exercise to facilitate smoking cessation, 12 trials comparing exercise groups to non-exercise groups were subjected to a meta-analysis (Figure 3). One trial of moderate quality could not be used for the analysis, as they did not report the number of participants, or proportion of the total number of participants in each group [49]. The meta-analysis showed that aerobic exercise did not significantly enhance the success rate of SC (Figure 3).

### 4.2. Effects of Exercise during Smoking Cessation Interventions on VO_2max_ and/or VO_2peak_

**Figure 4:**
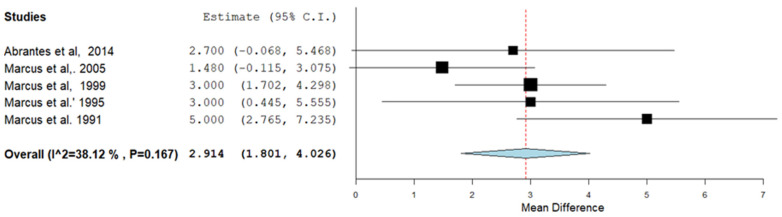
Forest plot for the trials on the effects of the intervention on maximal oxygen uptake (VO_2max_ and/or VO_2peak_). CI: confidence interval. The positive value of the ‘Mean Difference’ (*y*-axis) reflects a larger increase in VO_2max_ and/or VO_2peak_ (in mL/min/kg) in the exercisers compared to non-exercisers. References cited in the figure are: [40,41,45,46,47].

A meta-analysis of 5 trials (three high, one moderate and one low quality) [41,45,46,47] showed that aerobic exercise during smoking cessation interventions resulted in a higher VO_2max_ and/or VO_2peak_ than the other groups post intervention (Figure 4). No significant heterogeneity was found. The other trials were not included in the meta-analysis as they did not report mean and standard deviations for VO_2max_ and/or VO_2peak_ for each group.

## 5. Discussion

This review included a meta-analysis of 13 trials which assessed the effectiveness of aerobic exercise interventions on long-term SC and VO_2max_ and/or VO_2peak_. The main finding of this review was that there is no evidence that aerobic exercise enhances long-term SC. Nevertheless, aerobic exercise improved cardiopulmonary fitness in those who successfully quit smoking. The search identified no trials that assessed the effects of aerobic exercise on vaping cessation.

### 5.1. Design of the Exercise Studies and Verification of Smoking Cessation

Comparator groups received the same intervention as the exercise group, and consisted of face-to-face consultation [37,39,40,41,42,44,45,46,47,48,49], telephone counselling [41], behavioural treatment [43,48], nicotine gum [38,43], nicotine patch [48], inhalers [38], cognitive therapy [48], or combination of more than one treatment. In the trials included in the meta-analysis, smoking cessation was confirmed by measurement of the expired CO [38,39,40,41,42,43,44,46,47,48,49], saliva cotinine [40,45,46,47,48] or plasma thiocyanate [37] concentrations.

### 5.2. Exercise Interventions Do Not Enhance Smoking Cessation

When studying the benefits of exercise interventions for smoking cessation it is important to consider whether that is influenced by the frequency, intensity, time and type (FITT) of exercise [30,31,32].

Only two high quality trials reported that aerobic exercise intervention resulted in higher number of long-term successful quitters compared to other interventions [45,47]. These trials used 3 vigorous-intensity exercise sessions a week for 12–15 weeks. This is, however, an equivocal observation as three other high-quality trials with similar intensity, frequency and duration of exercise did not report a significant improvement in SC after aerobic exercise interventions [42,43,46]. As the effectiveness of exercise programs is highly dependent on adherence [50], it is possible that the benefits of exercise in two trials [45,47] and no benefits in another trial is related to the high adherence (68.7% and 88%, respectively), or low (55%) adherence [43] to the exercise interventions.

### 5.3. Exercise during Smoking Cessation Interventions Enhances VO_2max_ and/or VO_2peak_

Even if exercise does not benefit SC, there are substantial other benefits of exercise, such as the negative association with the prevalence of lung carcinoma in smokers and quitters [51] and a significant reduction in the mortality of smokers [52]. In addition, exercise during smoking cessation interventions led to a significant improvement in VO_2max_ and/or VO_2peak_ [40,41,42,44,45,46,47,48]. Improvements in VO_2max_ indicate improved aerobic exercise capacity and may also contribute to a reduction in the development of numerous clinical conditions and morbidities [53]. Besides these benefits for exercise capacity and diminishing the risk of future morbidity, there are also other physiological and psychological benefits to exercise as an adjunct to SC [54,55]. For example, exercise led to a reduction in withdrawal symptoms and improvement in psychological wellbeing, such as reduction in anxiety, depression and mood-swings [40,41,49]. Thus, even though exercise did not enhance the success rate of smoking cessation it nevertheless has significant beneficial effects for people seeking to stop smoking.

## 6. Vaping Cessation and Exercise

We were unable to locate any articles on the benefits of exercise for vaping cessation or improvement of VO_2max_ and/or VO_2peak_. However, given that the effects of smoking and vaping bear similarities [9,10] we expect that exercise will also have benefits for VO_2max_ and/or VO_2peak_ during vaping cessation.

## 7. Limitations

The low number of trials included in the meta-analysis on the effects of aerobic exercise on smoking cessation and cardiopulmonary fitness is a limitation in this review. In addition, this review excluded some special populations such as those suffering from asthma, COPD and/or pregnant women, in which exercise may enhance the success rate of smoking cessation. We also did not come across any studies on the benefits of exercise during vaping cessation.

## 8. Strengths

This review included only randomized control trials and used a rigorous tool to assess the quality of the trials (CROB2) to select best quality evidence. A Meta-analysis was conducted for both the effects of aerobic exercise on long-term SC and VO_2max_ and/or VO_2peak_. The review protocol was registered in PROSPERO database.

Future research is recommended to look at the effects of aerobic exercise on vaping cessation. Additionally, better quality of trial designs is recommended for future research. There is some evidence that supervised exercise sessions lead to a better rate of SC. We therefore suggest that further trials with supervised exercise sessions are warranted to investigate whether indeed supervised trails enhance the success rate of SC.

## 9. Conclusions

The meta-analysis showed no evidence that aerobic exercise promotes long-term smoking cessation. However, aerobic exercise improved VO_2max_ and/or VO_2peak_ and mental wellbeing in those who stopped smoking. The search identified no trials on the effects of aerobic exercise on vaping cessation. These observations encourage the inclusion of regular aerobic exercise in smoking- (and perhaps also vaping-) cessation programs.

## 10. Impact/Implication

This review suggests that aerobic exercise does not benefit the success of long-term smoking cessation. However, VO_2max_ and/or VO_2peak_ was improved in those who stopped smoking and will have a significant benefit for health and quality of life. It is therefore advisable to include aerobic exercise to any intervention for smoking cessation.

## Figures and Tables

**Table 1:** Keywords and search strategy used, using the PICOS approach in the selected databases.

	Population	Intervention	Comparison	Outcome Measures	Study Design
Search number and keywords	S1 = “smokers” “quit” OR “quitters” OR “smoking cessation” OR “stop smoking” OR “abstainers” OR “vape” OR “vaping” OR “e-cigarette” OR “e-cig” OR “electronic cigarette” OR “vapers” OR “e-cigarette users” OR “electronic cigarette users”	S2 = “cardiovascular exercise” OR “aerobic exercise” OR “aerobic training” OR “physical activity” OR “exercise” OR “physical exercise”	Interventions that include no aerobic exercise or structured changes in physical activity that are designed to support vaping or smoking cessation	S3 = “maximal oxygen uptake” OR “Exercise capacity” OR “carbon monoxide” OR “CO” OR “thiocyanate” OR “cotinine” OR “continuous abstinence” OR “continuous cessation” OR “prolonged abstinence” OR “prolonged cessation” OR “cessation” OR “stopping” OR “quitting”	Search was limited to randomised controlled trials (RCTs) to make a meta-analysis possible
Final search	Final search = S1 AND S2 AND S3				

**Table 3:** Results of the PEDro scale for quality assessment for the included randomised controlled trials.

Author (Year)	1. Eligibility Criteria Were Specified	2. Subjects Were Randomly Allocated to Groups	3. Allocation Was Concealed	4. The Groups Were Similar at Baseline Regarding Prognostic Indicators	5. There Was Blinding of All Subjects	6. There Was Blinding of All Therapists Who Administered the Therapy	7. There Was Blinding of All Assessors Who Measured at Least One Key Outcome	8. Measures of at Least One Key Outcome Were Obtained from More than 85% of the Subjects	9. All Subjects for Whom Outcome Measures Were Available Received the Treatment or Control Condition as Allocated	10. The Results of Between-Group Statistical Comparisons Are Reported for at Least One Key Outcome	11. Point Measures and Measures of Variability for at Least One Key Outcome Were Reported	Total PEDro Score
[39]	1	1	1	1	0	0	0	1	1	1	1	7
[41]	1	1	0	1	0	0	0	1	1	1	1	6
[38]	1	1	1	1	0	0	0	1	1	1	1	7
[44]	1	1	0	1	0	0	0	1	1	1	1	6
[48]	1	1	0	1	0	0	0	1	1	1	1	6
[40]	1	1	1	1	0	0	0	1	1	1	1	7
[47]	1	1	1	1	0	0	0	1	1	1	1	6
[46]	1	1	0	1	0	0	0	1	1	1	1	6
[43]	1	1	0	1	0	0	0	1	1	1	1	6
[45]	1	1	0	1	0	0	0	1	1	1	1	6
[49]	1	1	0	1	0	0	0	1	1	1	1	6
[37]	1	1	0	0	0	0	0	1	1	1	1	5
[42]	1	1	0	1	0	0	0	1	1	1	1	6

## Data Availability

Data is contained within the article.
